# Heart rate variability of elite female rowers in preparation for and during the national selection regattas: a pilot study on the relation to on water performance

**DOI:** 10.3389/fspor.2023.1245788

**Published:** 2023-08-24

**Authors:** Justin A. DeBlauw, Jesse A. Stein, Carolyn Blackman, Melissa Haas, Seraya Makle, Isis Echevarria, Rohan Edmonds, Stephen J. Ives

**Affiliations:** ^1^ Department of Health and Human Physiological Sciences, Skidmore College, Saratoga Springs, NY, United States; ^2^ Oak Ridge Institute for Science and Education, Belcamp, MD, United States; ^3^ United States Army Research Institute of Environmental Medicine, Natick, MA, United States; ^4^ Department of Health Sciences, Macquarie University, Sydney, NSW, Australia

**Keywords:** women, cardiac autonomic activity, rowing, peformance, longitudinal, training

## Abstract

Elite athletes require a delicate balance of physiological and psychological stress and recovery—essential for achieving optimal performance. Monitoring heart rate variability (HRV) provides a non-invasive estimation of both physiological and psychological stress levels, offering potentially valuable insights into health, performance, and adaptability. Previous studies, primarily conducted on male participants, have shown an association between HRV and performance in the context of rowing training. However, given the rigorous nature of rowing training, it is crucial to investigate HRV in elite rowers, particularly during the U.S. national selection regattas (NSR).

**Purpose:**

To comprehensively analyze elite female rowers, evaluating acute changes in HRV and subjective psychometrics during the NSR.

**Methods:**

Five elite female rowers (26 ± 2 years, 180 ± 8 cm, 82 ± 8 kg, 19 ± 6%fat) were recruited and tracked prior to and during NSR I and II. Morning HRV measures were completed using photoplethysmography (HRV4training) along with self-reported levels of fatigue, soreness, rating of perceived exertion, mentally energy and physical condition.

**Results:**

Significant decreases were observed in log transformed root-mean square of successive differences (LnRMSSD; *p* = 0.0014) and fatigue (*p* = 0.01) from pre-to-during NSR, while mental energy (*p* = 0.01), physical condition (*p* = 0.01), and motivation (*p* = 0.006) significantly increased. These psychometric measures returned to pre-NSR levels, at post-NSR (all *p* < 0.05), though HRV remained slightly suppressed. NSR on-water performance was not correlated to LnRMSSD or the change in LnRMSSD (*p* > 0.05).

**Discussion:**

HRV and psychometric measures are sensitive to the stress of elite rowing competition in females. However, HRV was not associated with on-water rowing performance during an elite rowing competition.

## 1. Introduction

In recent years, sport scientists and coaches have increasingly employed heart rate variability (HRV) as a non-invasive physiological marker for evaluating and enhancing athlete adaptations to training and subsequent performance (1–3). HRV serves as an index of the autonomic nervous system reflecting the interaction between the sympathetic and parasympathetic systems influence on the heart (4, 5). It is assessed by measuring the variation in R-R intervals, where a changes in the duration of R-R intervals indicates altered autonomic activity (3). It has previously been established that HRV measures are associated with health outcomes, adaptability to training regimens, and athletic performance (6–9), making HRV a good candidate for athlete monitoring. Intense training or psychological stress can suppress vagal indices of HRV, indicating reduced parasympathetic activity (7). Conversely, heightened sympathetic nervous system activity, as measured via HRV, has been linked to fatigue and overtraining (7, 8, 10). For athletes and coaches, it is important to understand and monitor the physiological and psychological stress associated with training, traveling, and competing, to optimize training and performance, but for some sports, such elite female rowers, there is a paucity of data.

Rowing is a high-intensity sport, which requires significant strength, power, anaerobic and aerobic capacity (11–14). International race distance is 2 km and which is covered in 5.5 to 7.5 min in elite rowers, depending upon boat class and gender (15) and course conditions (16). During racing, aerobic metabolism likely contributes 67%–84% of the energy requirement (15); thus, the remaining 16%–33% of energy demand is met through anaerobic energy producing pathways such as the phosphocreatine shuttle and anaerobic glycolysis (11, 17). Regarding the latter, peak rowing power over 5 or 10 stroke maximal tests has been found to be highly related to 500 m (18) and 2 km times (12). Rowing performance, both on the water and on a rowing ergometer, is dependent on several physical determinants, such as height and wing span [recently highlighted here (19)], and physiological determinants, as mentioned above, but also includes maximal oxygen consumption (VO_2max_) and the power output associated with VO_2max_ (*W*_VO2max_) (12, 20). However, quantifying on-water performance is often challenging because of the environment and the inability to assess the performance of the crew individually. Although ergometer performance tests eliminate these constraints, they do not sufficiently address the skill aspect of performance on the water (20), and can take away from technical training on the water which is also essential for performance. As such, identifying key physiological variables able to monitor and/or predict on-water performance has merit for coaches aiming to effectively periodize training workloads. Considering the rise in popularity of HRV monitoring tools for self-monitoring, in an autonomous manner, future studies should explore their real-world use.

Given the effectiveness of HRV as in indirect indicator of the body's ability to tolerate the stress of exercise training and the limited study of elite rowers, let alone female rowers, this pilot study sought to characterize a group of elite rowers (body composition, VO_2peak_, and peak rowing power output), and assess changes in autonomously monitored HRV and subjective psychometrics leading to, during, and shortly after U.S. National Selection Regattas (NSR). For this purpose, we hypothesized that HRV, self-reported levels of mental energy, and physical condition would significantly decrease during the NSR and recover following the competition rebound post-competition. It was also hypothesized that HRV, and the perturbation in HRV in response to NSR competition, would be related to on-water rowing performance. Additionally, we hypothesized that self-reported levels fatigue, soreness, rating of perceived exertion (RPE) would significantly increase during the NSR and return to normal post-competition.

## 2. Methods

### 2.1. Participants and general procedures

Elite female rowers were recruited from the Saratoga Rowing Association Advanced Rowing Initiative of the Northeast (ARION) program. The athletes were classified as elite development as they were training for national team selection and/or national and international level regattas, with rowing experience (secondary school and/or collegiate). Participants were recruited verbally and through emails and in coordination with the head coach. In addition to being part of the ARION training group, inclusion criteria required that the participants were healthy, English-speaking, and a smart-phone user. Exclusion criteria involved any chronic disease or illness or injury that would prevent them from training or one that could alter their HRV (e.g., atrial fibrillation), which was acquired by health history form and reviewed by the study team. The athletes provided written informed consent prior to participation. This protocol was reviewed and approved by the Skidmore College Institutional Review Board (IRB#2112-1010) and conducted in accordance with the most recent revisions to the Declaration of Helsinki.

### 2.2. Study overview

The athletes underwent in-person baseline testing in the Human Performance Research Laboratory at Skidmore College. Upon the participants' arrival, they were screened for eligibility and written informed consent was obtained. Baseline testing included a body composition analysis, a maximal power output test and a peak oxygen consumption (VO_2_peak) test (21–23). Following preliminary testing, participants tracked their HRV and psychometrics each morning leading up to and post the NSRs using the HRV4Training mobile device application. NSR 1 and NSR 2 occurred 5- and 9-weeks following baseline testing. The rowers official 2 km times during heats, semi-finals, and finals of the NSRs were used to assess the on the water performance, and the best times were used for analysis.

### 2.3. Baseline assessment

Height was measured using a stadiometer and body composition was obtained using air displacement plethysmography (Bod Pod, CosMed, Chicago, IL, USA) which is a known, reliable method of assessing body composition (22). To obtain the most accurate results possible, participants were asked to refrain from eating or exercise (only snack and water) at least 3 h prior to testing, use the restroom upon arrival, remove glasses and jewelry, if possible, to wear their uni-suit, and wore a provided swim cap to compress air pockets within the hair.

A maximal rowing power performance test was conducted during single trial following a participant controlled 5-min warm up (23). Starting from a rested (non-spinning) flywheel, participants were then instructed to row as hard as possible for 10 strokes completed (approximately 15 s) on a Concept2 rowing ergometer (Model D, Concept2, Morrisville, VT) a pre-determined drag factor of 10, as described previously (18, 24). The maximum, or highest, single power output in watts was recorded. A brief recovery was allotted, and then a graded VO_2peak_ test was run to characterize the aerobic fitness of the rowers (15). To measure oxygen consumption, participants were instrumented with a two-way non-rebreathe mouthpiece (8,900, HansRudolph, Shawnee, KS) attached via hose to a metabolic cart (TrueOne 2,400, Parvomedics, Sandy, UT), which has been documented to be reliable and valid assessment of VO_2_ (21). The participant also wore a chest strap style heart rate monitor (H7, PolarUSA, Lake Success, NY). Participants were seated on the rowing machine until reasonable baseline relative VO_2_ values were obtained (3–5 ml/kg/min) before starting the graded exercise test. The graded exercise test started at 120 watts for 3 min, increasing 30 watts per 3 min stage until volitional exhaustion and/or failure to maintain workload following a similar previously published protocol (25). Increases in work rate were achieved through increased drive force (“pressure”) and/or stroke rate (strokes/minute). This was a maximal effort test. Participants' HR, ventilation, VO_2_, and respiratory exchange ratio (RER) were continuously monitored throughout the test and as criteria as to whether participants achieved a near-maximal or maximal VO_2_.

### 2.4. HRV and psychometric monitoring

Following baseline testing participants were tasked with remotely recording their heart rate variability each morning upon waking using the HRV4Training mobile device application (Amsterdam, Netherlands; see http://www.hrv4training.com/). The HRV4Training application uses photoplethysmography (PPG) to obtain R-R intervals from a continuous pulse rate reading (26, 27). Participants were given a familiarization session with the application along with an instructional document for reference; additionally, the application uses a step-by-step process to walk the user through the measurement. In the morning after voiding their bladder, participants recorded their HRV for 1 min upon waking while in a supine position while breathing at self-selected pace (28). HRV was tracked using root mean square of successive differences (RMSSD), natural log transformed RMSSD (LnRMSSD), and standard deviation of N-N intervals (SDNN). The mobile device application also tracks other factors that may influence HRV, such as sleep, training, menstrual cycle status, which the participant was asked to complete with each reading. A two-week period was used to establish a normal range for each individual, the app provided a daily assessment as to whether the athletes' HRV was within, above, or below their normal range. This data was relevant for comparing HRV and training performance.

### 2.5. Data analysis

During the weeks of March 22nd–25th the participants competed in NSR I and May 3rd–6th in NSR II. The rower's 2 km performance times were recorded and compared to their HRV of the corresponding day. Additionally, changes in LnRMMSD, 7-day average of LnRMMSD, RHR, SDNN, RPE, mental energy, soreness, fatigue, physical condition, and motivation pre, during, and post-NSR were assessed with a linear mixed model with the fixed effect of time (pre, during, post NSR) and the random effect of subject ID. Significant main effects were followed up by pairwise comparisons. Linear mixed models were checked for homoscedasticity via visual inspection and Q-Q plots. Finally, a linear regression was performed to assess the relationship between best on-water performance, absolute LnRMMSD during the each NSR, and the percent change in LnRMMSD from pre to during NSR. All calculations and statistical analyses were run using Microsoft Excel (Microsoft Excel, v 16.43, Redmond, United States) and open-source statistical software JAMOVI (29, 30). Estimates of effect size, using Cohen's small (0.2), medium (0.5), and large (0.8) were used in accordance with the model complement *p* values. The *α*-level was set to 0.05 and used to determine statistical significance.

## 3. Results

### 3.1. Baseline subject characteristics and performance

Participants were 26.4 ± 1.7 years, height 180 ± 2 cm, weight 81.8 ± 8.4 kg, percent body fat 19.3 ± 6.5%, and percent fat free mass 80.7 ± 6.5% (Table 1). Participants baseline and on water performance characteristics are presented in Table 2, average maximal power output was 681 ± 71 watts, peak heart rate 181 ± 17 bpm, and the relative VO_2peak_ 52.6 ± 3.4 ml/kg/min (Table 2). All participants VO_2_ max ranked >75th percentile for age and sex specific norms. Individual daily HRV values and rolling averages are presented in Table 3.

**Table 1:** Subject age, height, weight, % fat and % fat free mass.

Subject		Height	Weight		% fat free
ID	Age	(cm)	(kg)	% fat	Mass
1	29	184	75.0	11.1	88.9
2	25	177.5	73.0	18.8	81.2
3	27	179	93.0	26.0	74.0
4	27	179	90.8	27.2	72.8
5	24	179	77.3	13.4	86.6
Mean ± SD	26.4 ± 1.74	179.7 ± 2.23	81.8 ± 8.39	19.3 ± 6.47	80.7 ± 6.48

**Table 2:** Subject laboratory and on water performance characteristics.

ID	Peak power (W)	Peak HR (bpm)	Peak absolute VO_2_ (L/min)	Peak relative VO_2_ (ml/kg/min)	Age adjusted VO_2_ ranking	Anaerobic threshold (AnT) (W)	HR at AnT (bpm)	Average NSR 1 time (s)	Average NSR 2 time (s)
1	643	184	4.22	56.3	95th %	270	173	452.64	427.01
2	571	195	4.16	57.06	95th %	240	181	461.99	425.31
3	763	190	4.64	49.89	75–80th %	240	165	445.64	N/A
4	677	188	4.49	49.44	75–90th %	240	159	469.27	435
5	750	183	3.89	50.34	75–90th %	240	168	461.00	429.71
Mean ± SD	681 ± 71	181 ± 17	4.3 ± 0.3	52.6 ± 3.4	N/A	246 ± 12	169 ± 7		

**Table 3:** Individual daily and rolling 7-day average of LnRMSSD pre, during, and post NSR 1 &2.

ID	Pre-NSR 1 daily LnRMMSD							During NSR 1 daily LnRMMSD				Post-NSR 1 daily LnRMSSD						
1	8.0	7.4	8.0	8.4	7.6	8.4	8.0	7.8	7.7	7.6	7.4	6.6	7.7	7.6	8.0	8.7	9.2	8.3
2	7.2	8.2	7.0	7.2	7.0	7.6	7.2	7.5	7.2	6.8	7.0	7.5	7.0	7.8	8.0	7.2	7.9	7.5
3	8.1	8.4	8.7	9.1	8.6	8.6	8.4	–	–	–	–	–	–	7.6	8.3	–	8.2	8.8
4	7.8	8.3	9.0	7.6	9.1	8.4	7.2	7.7	7.7	8.1	6.4	8.1	–	8.7	7.6	7.3	7.4	7.3
5	8.9	–	10.2	8.9	10.9	12.6	8.7	–	–	9.0	9.6	8.7	9.8	8.4	8.4	8.9	8.3	–
ID	Pre-NSR 2 daily LnRMMSD							During NSR 2 daily LnRMMSD				Post-NSR 2 daily LnRMSSD						
1	7.9	7.6	9.4	7.9	–	9.6	9.0	8.4	8.1	7.9	8.0	7.7	9.2	8.8	8.2	7.3	7.5	7.1
2	7.1	7.1	7.9	7.6	7.4	8.0	7.3	7.2	7.6	6.9	7.1	8.2	8.7	7.7	7.7	7.6	7.2	7.7
3	-	9.0	–	8.7	–	7.0	8.1	–	8.8	–	9.0	8.8	7.8	8.8	8.6	8.8	8.5	8.4
4	7.5	7.2	7.4	7.5	8.4	8.2	7.2	6.3	6.2	8.1	7.8	7.3	6.3	7.2	-	8.2	8.0	8.7
5	9.6	–	–	9.2	7.8	9.3	9.6	–	8.7	9.1	8.6	8.6	–	9.0	9.2	8.7	9.2	8.6
ID	Pre-NSR 1 7-day average LnRMSSD							During NSR 1 7-day average LnRMSSD				Post-NSR 1 7-day average LnRMSSD						
1	8.3	8.2	8.1	8.2	8.0	8.1	8.0	8.0	8.0	7.9	7.8	7.6	7.5	7.5	7.5	7.6	7.9	8.0
2	7.3	7.5	7.4	7.5	7.4	7.4	7.3	7.4	7.2	7.2	7.2	7.3	7.2	7.3	7.4	7.3	7.5	7.6
3	8.2	8.2	8.3	8.5	8.5	8.6	8.6	NA	NA	NA	NA	8.5	8.4	7.6	8.0	8.0	8.1	8.2
4	7.7	7.8	7.9	7.9	8.2	8.3	8.2	8.2	8.1	8.0	7.8	7.6	7.5	7.8	7.8	7.7	7.6	7.7
5	9.6	9.5	9.7	9.5	10.0	10.4	10.2	10.3	10.0	10.2	9.7	9.2	9.1	9.0	9.0	8.9	7.5	7.6
ID	Pre-NSR 2 7-day average LnRMSSD							During NSR 2 7-day average LnRMSSD				Post-NSR 2 7-day average LnRMSSD						
1	7.7	7.7	7.9	7.9	7.9	8.2	8.5	8.6	8.7	8.4	8.5	8.4	8.3	8.3	8.3	8.2	8.1	8.0
2	7.3	7.2	7.4	7.4	7.4	7.5	7.5	7.5	7.5	7.4	7.3	7.4	7.6	7.6	7.7	7.7	7.7	7.8
3	8.3	8.4	8.7	8.6	8.6	8.3	8.2	8.2	8.2	8.2	8.2	8.3	8.5	8.7	8.6	8.6	8.6	8.5
4	7.5	7.2	7.4	7.5	8.4	8.2	7.2	6.3	6.2	8.1	7.8	7.3	6.3	7.2	–	8.2	8.0	8.7
5	8.6	8.6	8.6	8.7	8.5	8.9	9.1	9.0	8.9	8.9	8.8	9.0	8.9	8.8	8.9	8.9	8.9	8.9

### 3.2. Changes in estimated cardiac autonomic activity during the NSR

**Figure 1:**
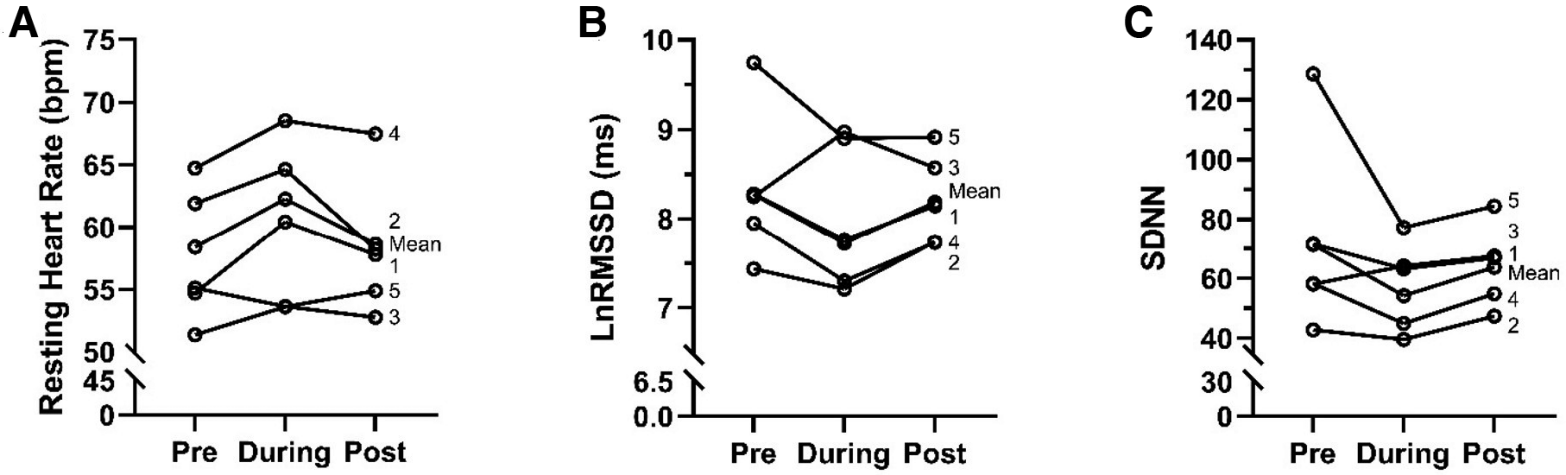
Estimated cardiac autonomic assessments pre, during, and post NSRs in elite female rowers (*n* = 5). (**A**) Resting heart rate, RHR (beats/min), (**B**) natural log transformed root mean square of successive differences, LnRMMSD, (**C**) standard deviation of N-N intervals, SDNN.

A significant main effect of time was observed in LnRMSSD (*F* = 3.82, *p* = 0.02, Figure 1A). A pairwise comparison revealed a significant reduction in LnRMSSD occurred for pre-to-during NSRs (*p* = 0.006, *t* = 2.78, ES = 0.47). LnRMSSD was not different during-to-post NSRs (*p* = 0.15, *t* = 1.87, ES = 0.24) or pre-to-post NSRs (*p* = 0.11, *t* = −1.61, ES = 0.27). There was a significant main effect for time on RHR (*F* = 5.51, *p* = 0.005, Figure 1B). A pairwise comparison revealed a significant increase in RHR from pre-to-during NSRs (*p* = 0.001, *t* = −3.32, ES = 0.56) and from during-to-post NSRs (*p* = 0.02, *t* = 2.25, ES = 0.38). There was no significant difference in RHR from pre-to-post NSRs (*p* = 0.19, *t* = −1.32, ES = 0.22) Additionally, there was no main effect for time on SDNN (*F* = 2.91, *p* = 0.05, Figure 1C) during the NSRs. As an aspect of the current study was to utilize autonomous HRV monitoring (vs. laboratory and/or researcher based) we observed a 91% compliance rate, with the athletes completing 141 out of 154 possible measurements over the study time frame.

### 3.3. Changes in psychometric dimensions of performance during the NSR

**Figure 2:**
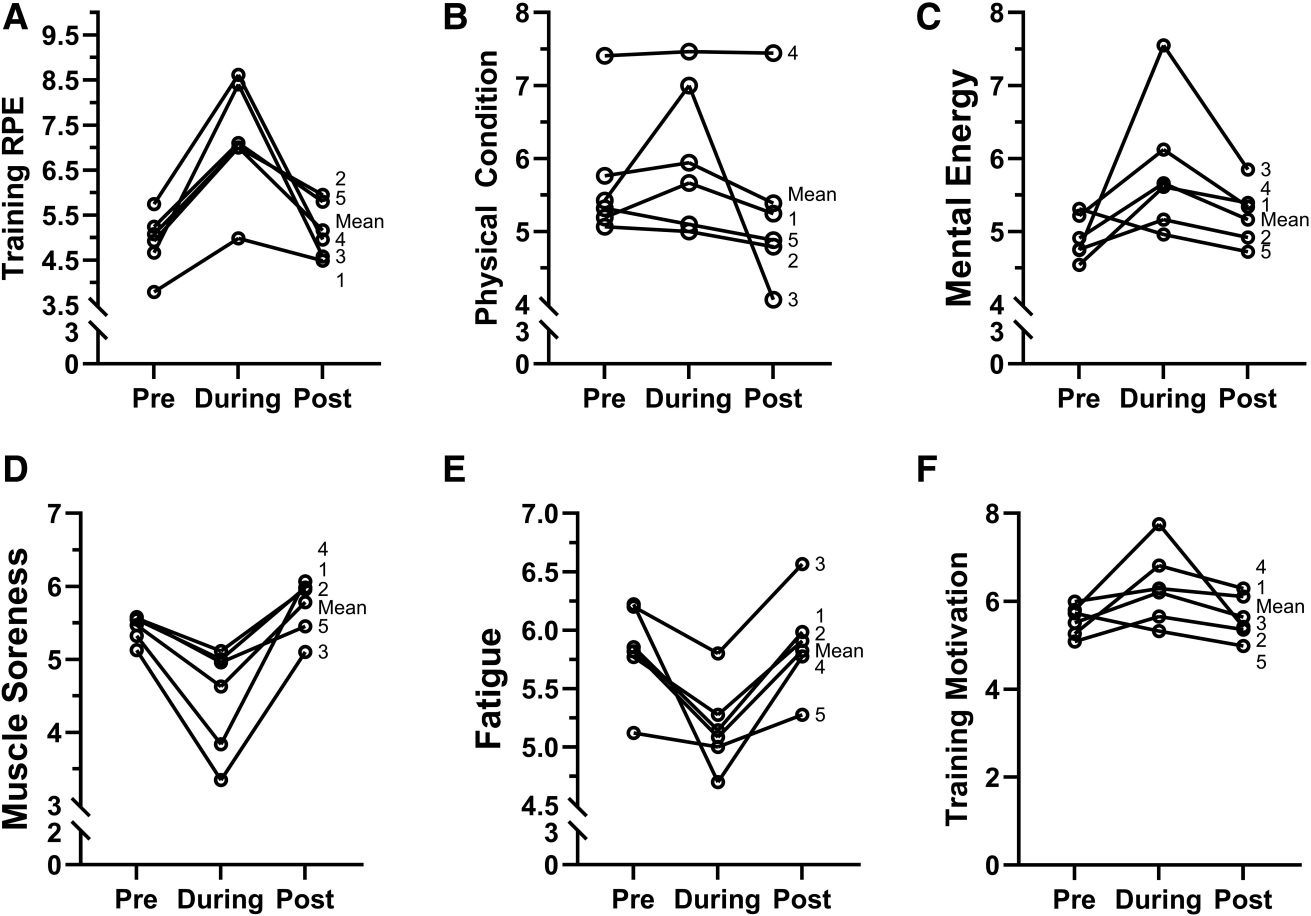
Performance related psychometrics for pre, during, and post NSRs in elite female rowers (*n* = 5). (**A**) Training rating of perceived exertion (training RPE), (**B**) physical condition (**C**) mental energy, (**D**) muscle soreness, (**E**) fatigue, (**F**) training motivation.

A significant main effect of time was observed for mental energy (*F* = 5.05, *p* = 0.008, Figure 2C). A pairwise comparison revealed a significant increase in mental energy for pre-to-during NSRs (*p* = 0.002, *t* = −3.17, ES = 0.53) followed by a significant decrease for during-to-post NSRs (*p* = 0.03, *t* = 2.20, ES = 0.37). Pairwise comparison revealed no significant difference in mental energy between pre-to-post NSRs (*p* = 0.23, *t* = −1.19, ES = 0.20). A significant main effect of time was observed for soreness (*F* = 10.4, *p* = 0.001, Figure 2D). A pairwise comparison revealed that soreness was significantly decreased for between pre-to-during NSRs (*p* = 0.001, *t* = 3.27, ES = 0.55) and a significant increase for during-to-post NSRs (*p* = 0.001, *t* = −4.52, ES = 0.76). No significant difference was observed for pre-to-post NSRs (*p* = 0.15, *t* = −1.44, ES = 0.24). A significant main effect of time was observed for fatigue (*F* = 8.03, *p* = 0.001, Fatigue 2E). A pairwise comparison revealed a significant decrease in fatigue for pre-to-during NSRs (*p* = 0.01, *t* = 3.49, ES = 0.59), while a significant increase in fatigue was observed for during-to-post NSRs (*p* = 0.001, *t* = −3.72, ES = 0.63). There was no significant difference in fatigue for pre-to-post NSRs (*p* = 0.79, *t* = −0.26, ES = 0.04). A significant main effect for time was found for physical condition (*F* = 4.84, *p* = 0.009, Figure 2B). Additionally, a pairwise comparison found no change in physical condition for pre-to-during NSRs (*p* = 0.21, *t* = −1.26, ES = 0.21). While a significant decrease was observed for during-to-post NSRs (*p* = 0.003, *t* = 2.99.56, ES = 0.50) and between pre-to-post NSRs (*p* = 0.04, *t* = 2.03, ES = 0. 34). A significant main effect for time was observed for motivation (*F* = 6.05, *p* = 0.003, Figure 2F). A pairwise comparison found a significant increase in motivation for pre-to-during NSRs (*p* = 0.001, *t* = −3.32, ES = 0.56), while a significant decrease in motivation was observed for during-to-post NSRs (*p* = 0.004, *t* = 2.92, ES = 0.49). No significant differences in motivation were observed for pre-to-post NSRs (*p* = 0.61, *t* = −0.50, ES = 0.08). A significant main effect was observed for training RPE (*F* = 13.4, *p* = 0.001, Figure 2A). A pairwise comparison found a significant increase in training RPE for pre-to-during NSRs (*p* = 0.001, *t* = −4.99, ES = 0.91) and a significant decrease from during-to-post NSRs (*p* = 0.001, *t* = 4.15, ES = 0.76). No significant differences in training RPE for pre-to-post NSRs (*p* = 0.43, *t* = −0.78, ES = 0.14).

### 3.4. Estimated cardiac autonomic activity and relation to on-water rowing performance

**Figure 3:**
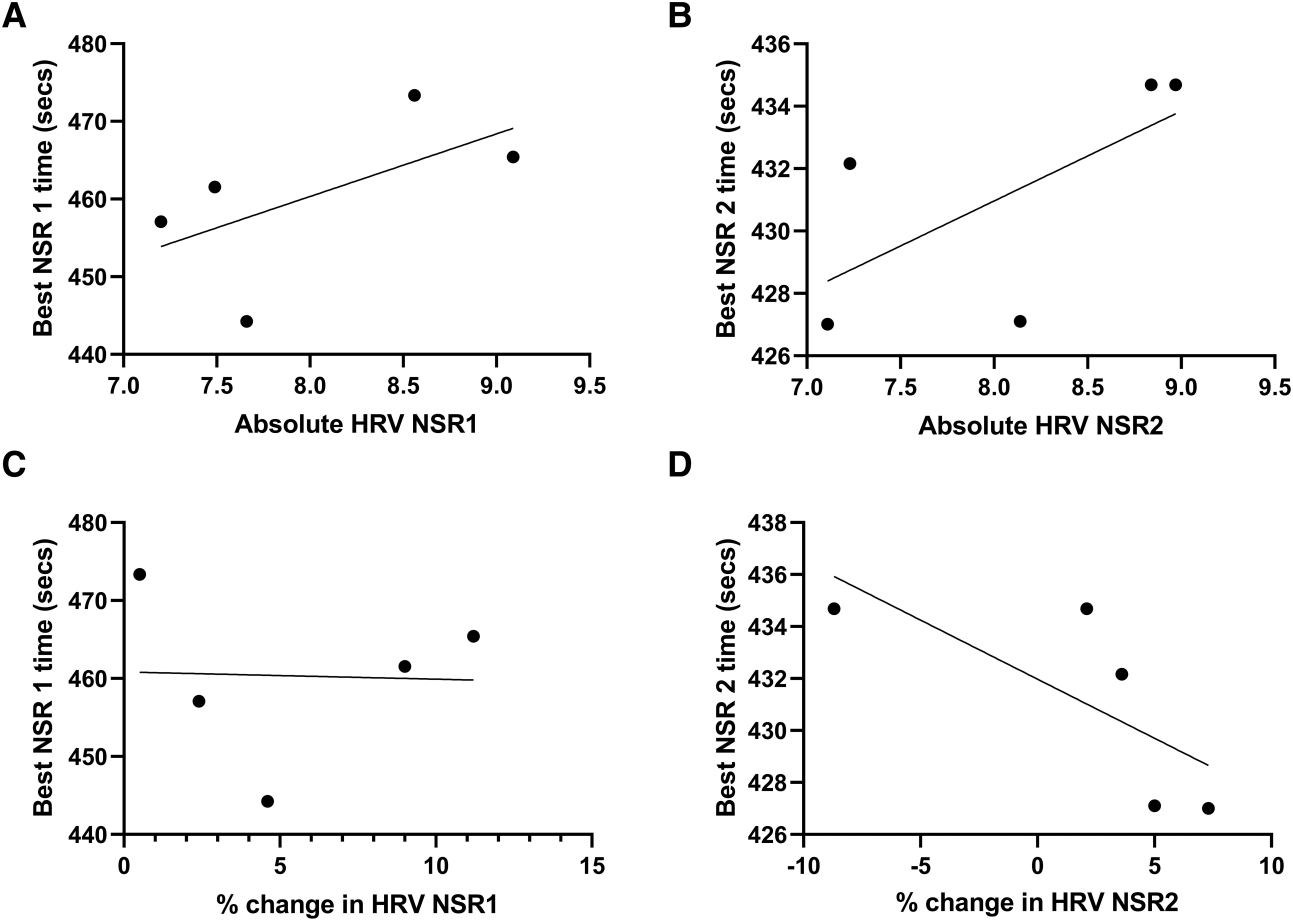
The relationship between on-water performance and average absolute LnRMMSD and % change in LnRMMSD during NSR 1 & 2 in elite female rowers (*n* = 5). (**A**) Absolute HRV (LnRMSSD) and best NSR 1 performance, (**B**) absolute HRV (LnRMSSD) and best NSR 2 performance, (**C**) % change in HRV (LnRMSSD) and best NSR 1 performance, (**D**) % change in HRV (LnRMSSD) and best NSR 2 performance.

No significant relationship was observed for LnRMSSD during the NSR 1 and on water performance (*p* = 0.29, R = 0.59, *R*^2^ = 0.35, Figure 3A) or for the % change in LnRMMSD from pre to during NSR 1 and on water performance (*p* = 0.95, R = 0.03, *R*^2^ = 0.00, Figure 3C). Additionally, no significant relationship was observed for LnRMMSD during NSR 2 and on water performance (*p* = 0.23, R = 0.65, *R*^2^ = 0.42, Figure 3B) or for the % change in LnRMMSD from pre to during NSR 2 and on water performance (*p* = 0.16, R = 0.73, *R*^2^ = 0.53, Figure 3D).

## 4. Discussion

The current pilot study was one of the first to assess the autonomously measured changes in HRV and psychometrics during high-level competition and relation to on-water performance in elite female rowers. The purpose of the current study was to characterize a group of elite rowers (body composition, peak aerobic fitness, and maximal power output), and assess acute changes in HRV, as an estimate of cardiac autonomic activity, and subjective psychometrics during the NSRs. We observed decreases in LnRMSSD during competition which rebounded 72 h post competition. However, neither HRV nor the change in LnRMSSD were related to on-water performance during the NSR. Secondly, these changes were accompanied by alterations in psychometrics, such increased mental energy, decrease in fatigue, and increased self-report of physical condition, while soreness was unaffected during the competition; these parameters returned to baseline approximately 72 h post competition. This is the first study to report autonomous HRV and perceptual dimensions of training and performance in rowers; thus, collectively we demonstrate that athletes are capable, and willing, to self-monitor (observed 91% compliance rate), which can provide an insight into their physiological and psychological status in the context of high-level competitions.

### 4.1. Changes in estimated cardiac autonomic activity during the NSRs

We observed significant acute decreases in LnRMSSD from pre to during the NSRs with a rebound occurring 72 h post NSR (Figure 1). An oversimplification or lack of context to this response could be a cause for concern since decreases in HRV during a training cycle are often interpreted as indicative of a lack of adaptability to training (31), and when depressed chronically, LnRMMSD is related to all-cause mortality (32, 33). The observed acute decrease in HRV can be attributed to pre-performance anxiety or stress response as previous research has demonstrated an anticipatory response to stressful tasks, meaningful competitions and high-intensity training sessions decreases HRV (34–36). The performance impact of HRV reductions are equivocal, and could be attributed to the level of athlete and/or the importance of the forthcoming performance bout (37–39). As such, these acute changes can be seen as expected or beneficial, as increases in sympathetic activity facilitate increases in norepinephrine, epinephrine (36), bioenergetic pathways (40, 41), and neurological processes (i.e., reaction time) (42). Thus, this short-term suppression of HRV estimated cardiac autonomic activity may indicate a “readiness to perform”, as indicated in previous literature (43). Our observation that parasympathetic reactivation did not occur within 72 h partially contradicts previous literature where an observed rebound occurred within 48 h (39, 44). This difference might be attributed to the effects of a multiday event delaying full autonomic recovery, and/or the potential for travel to suppress HRV (45). Therefore, athletes, coaches and or sport scientists using HRV monitoring strategies should consider adopting an individualized return to training plan.

### 4.2. Changes in psychometric dimensions of performance during the NSRs

This study found that psychometric profiles improved during NSRs and returned to pre-NSR values following the competition (Figure 2). Specifically, we found improvements in mental energy, physical condition, motivation, in addition to the reductions in fatigue occurred during competition as LnRMSSD decreased. Flatt et al., (2017b) observed that both psychological (stress, soreness, fatigue, and mood) and LnRMMSD profiles improved (i.e., LnRMMSD increased and perceived fatigue decreased) as Division I swimmers tapered for a national competition. The improvements in psychological and physiological status when reducing training volume likely contribute to the ergogenic effects associated with tapering (46). Similarly, our group has demonstrated significant reductions in fatigue and improvements in motivation in recreationally active volunteers participating in HRV-modulated training prescribed with lower training volumes (47). Reductions in LnRMSSD have previously been associated with reductions in fatigue and soreness (48); however, these findings are not universal and may be subject to interindividual variability associated with HRV outcomes (49). While reductions in LnRMSSD have been associated with increased training stress and incomplete recovery (50) the changes in LnRMSSD are interpreted as a positive response due to the increases in psychological status, which other investigators have suggested are key to interpreting HRV outcomes (51). Thus, the inclusion of perceptual or psychometric assessments is valuable to coaches, athletes, and sports scientists monitoring athlete workload.

### 4.3. Estimated cardiac autonomic activity and on-water performance during the NSRs

This study found that neither absolute LnRMMSD or the % change in LnRMMSD during competition was related to on-water performance. This finding supports the previous research by DeBlauw et al. (2022), that found no difference in 40-min cycling time trial performance when HRV (LnRMMSD) was within or outside an individual's smallest worthwhile change window. There is substantial evidence that daily HRV monitoring is a useful tool for monitoring and adapting training cycles (52–54) as well as identifying when negative adaptations or overtraining may be present (8). However, HRV's relation to performance may be more nuanced than initially thought, in that parasympathetic (LnRMSSD) HRV metrics suggest that an inverse relation may exist insofar as greater HRV may actually be associated with slower on water racing times (R = 0.6–0.7, Figure 3), and the percent change in the same metric is unrelated to on water performance. Thus, rowers and/or coaches may consider hiding or masking HRV values during competition to avoid further anxiety around their values and interpretation.

### 4.4. Experimental considerations

The sample size used in the current study was small but was representative of female rowers from an elite team. No sample size estimation or power analysis was conducted because we aimed to recruit all of the rowers on the team who were currently at the training center. Due to intensive training camp logistics we were only able to recruit 5 athletes for this study, similar to other investigations ranging from 2 to 6 elite athletes (Edmonds et al., 2014; Plews et al, 2012). Due to the remote nature of the HRV data collection we were unable to enforce a pre-reading stabilization period or seated position for HRV readings which may have an effect on resting HRV values (27, 55). Additionally, the statistical analysis used in our previous investigations demonstrated that changes in HRV is an appropriate method in similar small populations (56). Finally, we did not control for the menstrual cycle phase in this study. It is important to note that (57) has demonstrated that in female rowers large perturbations in autonomic activity can occur during the menses phase of the ovarian cycle, which may overlap with competition, and this did occur within our one of our specific athletes. This may have influenced their respective LnRMMSD values. However, these athletes will still be expected by coaches and themselves to perform at high-level regardless of menses. Despite this, our approach represents an ecologically valid perspective given that these women likely train and compete during all phases of the menstrual cycle. Further research may be necessary to determine more significant relationships and findings, but the current study provides effect sizes for subsequent studies and meta-analyses.

### 4.5. Future directions

There has been limited previous literature on how HRV may affect water performance for rowers, especially female populations. The importance of assessing HRV in an underrepresented population may provide extremely useful feedback for coaches when designing an effective exercise program. Further applications on HRV could provide a stronger or more significant relationships of HRV and on water performance. Further assessment of an individual's HRV within and outside of their normal window (above, below and normal ranges) and the effect on rowing performance.

## 5. Conclusion

The current pilot study is one of the first to use autonomous monitoring of HRV and psychometrics in elite female rowers leading up to, during, and following U.S. National Selection Regattas, and characterized their performance related parameters (body composition, peak aerobic fitness, and maximal power output). We observed decreases in LnRMSSD during competition, which was mirrored by increased mental energy, decrease in fatigue, and increased self-report of physical condition, which reversed 72 h post competition. However, neither HRV nor the change in LnRMSSD were related to on-water performance during the NSR, perhaps the reductions in HRV are reflecting a readiness to perform and not an acute maladaptive response. Collectively, this novel pilot study highlights that athletes are capable of autonomous monitoring HRV and perceptual dimensions of training and performance; which can provide an insight into their physiological and psychological state but the temporal relations amongst these variables may be complex and context dependent.

## Data Availability

The raw data supporting the conclusions of this article will be made available by the authors, without undue reservation.
